# Carbon Monoxide Poisoning in Kuwait: A Five-Year, Retrospective, Epidemiological Study

**DOI:** 10.3390/ijerph18168854

**Published:** 2021-08-22

**Authors:** Abdullah Al-Matrouk, Ali Al-Hemoud, Mohammed Al-Hasan, Yaqoub Alabouh, Amal Dashti, Haider Bojbarah

**Affiliations:** 1 Narcotic and Psychotropic Laboratory, Department of Criminal Evidence, Ministry of Interior, Al-Dhajeej, Al-Farwaniya 85000, Kuwait; akalmatrouk@moi.gov.kw (A.A.-M.); amaldashti@moi.gov.kw (A.D.); hydene67@yahoo.com (H.B.); 2 Environment and Life Sciences Research Center, Kuwait Institute for Scientific Research, P.O. Box 24885, Safat 13109, Kuwait; 3 Toxicology Laboratory, Department of Criminal Evidence, Ministry of Interior, Al-Dhajeej, Al-Farwaniya 85000, Kuwait; m_alhasan@live.com; 4 Faculty of Dentistry, Kuwait University, P.O. Box 24923, Safat 13110, Kuwait; yaqoub.alabwah@ku.edu.kw

**Keywords:** carbon monoxide poisoning, carboxyhemoglobin, CO-oximetry, Kuwait

## Abstract

Background: Carbon monoxide (CO) poisoning is a major public health concern and a common cause of death worldwide. However, to our knowledge, no studies have been conducted on CO poisoning exposure and mortality in Kuwait. Objectives: Using epidemiological and forensic data analysis, we investigated the prevalence and characteristics of CO poisoning-associated deaths in Kuwait over five years (2014–2018), using official police data. Methods: The Forensic Toxicology Laboratory analyzed 203 blood specimens of deceased individuals for potential CO poisoning during the study period. We obtained demographic information of the deceased and other information regarding the source of the CO, the type of death and the seasonal and geographical distribution of fatalities. The percentage of carboxyhemoglobin (COHb%) was assessed using a CO-oximeter. Results: CO poisoning was confirmed in ~29% (59 cases) of the analyzed specimens, of which CO poisoning was accidental in the majority of cases (~95%) and homicidal in the remaining of cases (~5%), with no reported suicides. The five-year cumulative mean of COHb% in the blood specimens of the 59 confirmed cases was ~63%. Most of the deceased were males (~68%). The mean age of male and female victims per year were similar (~23–38 years). Fatalities were more common (~44%) during the winter (December–February). Uncontrolled home fires and coal stoves contributed to the primary sources of CO poisoning at 61% and 22%, respectively. Recommendations: Based on our findings, we propose that the local government should mandate the installation of smoke alarms and CO detectors in residential settings and endorse health education, informing the local population about the causes of fire and potential for CO poisoning, with an emphasis on prevention. Practical measures that can be applied include proper installation and regular maintenance of home-heating appliances and appropriate ventilation. The present study could greatly benefit the government in directing efforts toward decreasing CO poisoning incidents in Kuwait in the future.

## 1. Introduction

Carbon monoxide (CO) is one of the leading causes of death by poisoning worldwide and is associated with approximately 4.6 deaths per million individuals [1]. It is a colorless, tasteless, non-irritant, toxic gas produced both endogenously and exogenously [2]. CO is naturally present in the atmosphere at a low concentration, being approximately 0.03–0.20 parts per million (ppm) [1]. Endogenously, it is produced during the metabolism of hemoproteins within the body. Exogenously, it is a by-product of incomplete combustion of carbonaceous compounds from sources such as vehicle exhaust, fires and improperly maintained heating systems [2,3]. The World Health Organization recommends that the average exposure to CO should not exceed 87, 52, 26, 9 and 6 ppm for periods of 15 min, 30 min, 1 h, 8 h and 24 h, respectively [4].

CO is known as the “silent killer”, since it is challenging to detect until symptoms of CO poisoning develop [5]. Poisoning starts with the inhalation of a relatively high concentration of CO gas, which is rapidly absorbed through the lungs and diffuses across the alveolar and capillary membranes [1]. Once absorbed, it binds to the heme groups of hemoproteins, including hemoglobin (Hb), myoglobin and cytochrome c oxidase (COX). The relative affinity of Hb for CO is much higher than that of myoglobin and COX. CO binds to Hb in the blood, forming a complex known as carboxyhemoglobin (COHb) [3].

The binding of CO to the heme moiety in hemoproteins alters their biochemical functions, such as the ability to carry and reduce oxygen or transfer electrons [6]. The pathophysiology of CO poisoning includes (1) a reduction in the global oxygen supply, (2) inhibition of mitochondrial respiration and (3) activation of inflammatory signaling pathways [3].

The clinical manifestations of CO intoxication are often nonspecific and depend on several factors, including (1) the concentration of inspired CO, (2) the duration of exposure and (3) the overall health status of the individual (pulmonary ventilation, physical condition and the rate and efficiency of breathing) [2]. The average blood concentration of COHb varies among individuals. On the one hand, a healthy, non-smoking person has a COHb level of 2% or less. Based on national health and nutrition surveys, the COHb level among participants averaged 0.83 ± 0.67% among never smokers (50th and 95th percentiles were 0.72% and 1.65%, respectively) [7,8]. On the other hand, the level of COHb in heavy smokers is rarely above 10% [7]. CO poisoning is asymptomatic at a blood concentration of less than 10%; at levels of 10% or greater, neurological symptoms such as nausea, headache and dizziness develop. When the average blood concentration of COHb reaches 30–50%, increases in respiratory and heart rates, syncope, motor paralysis and confusion are observed. Beyond 50%, COHb is considered life-threatening [2]. Symptoms associated with CO poisoning, in ascending order of severity, are (1) mild headache, fatigue, nausea, vomiting, dizziness and blurred vision, (2) confusion, chest pain, dyspnea, weakness and tachycardia and (3) palpitations, cardiac dysrhythmias, hypotension, myocardial ischemia, cardiac arrest, respiratory arrest, pulmonary edema, seizures and coma [9].

Globally, mortality associated with CO poisoning is mostly attributed to fires for heating; however, approximately a third of deaths are attributed to stoves, portable heaters and automobile exhausts, either due to an obstruction or malfunctioning of exhaust systems or attempted suicide [10]. In the Gulf Cooperation Council region, conditions cold enough to prompt the use of heating systems occur infrequently and only during the winter months. A study conducted in the United Arab Emirates revealed that burning charcoal in poorly ventilated rooms during the winter months was the primary source of CO poisoning [11]. Similarly, Alberreet et al. (2019) reported that CO poisoning events in the Middle East and north Africa occurred in the winter, in which gas heaters were the most frequent source [12]. According to that study, the majority (~98%) of cases were accidental and a minority (~2%) were intentional (either suicidal or homicidal). A ten-year study (2004–2013) conducted in Saudi Arabia using autopsy reports revealed that ~8% of the deceased were diagnosed with acute CO toxicity [13]. Most of the incidents (~91%) were accidental, with there being more males than female victims. The highest rate was in the winter months (50%) and 40–50-year-olds were disproportionately affected, followed by 31–40-year-olds.

CO poisoning is a common incident worldwide, but knowledge regarding its epidemiology in Kuwait is insufficient. Therefore, this study was conducted to investigate the prevalence and characteristics of CO poisoning in Kuwait using samples and data obtained from the Toxicology Laboratory and Forensic Medicine Division of the General Department of Criminal Evidence, Kuwait.

## 2. Materials and Methods

The data used in this study were obtained from the General Department of Criminal Evidence of Kuwait, using samples collected and analyzed from 2014 to 2018 by the Departments of Forensic Pathology and Forensic Toxicology. The study was authorized by the Ministries of Justice and the Interior (reference number 221232). Specimen collection and analysis were authorized by the Ministries of Justice and the Interior. Consent was obtained from the legal guardians or family members of the deceased subjects.

The Forensic Toxicology Laboratory analyzed 203 blood specimens of deceased individuals for potential CO poisoning during the study period. Analytical reports were provided to the Forensic Pathology Unit to determine the cause of death; COHb levels of more than 50% were considered as confirmed CO poisoning. We collected and analyzed all laboratory reports that were generated for individuals suspected of having died from CO poisoning from 2014 to 2018. COHb levels were measured by the Forensic Toxicology Laboratory only when CO poisoning was suspected. Information about the deceased was gathered from the Forensic Pathology Unit, although certain details were inaccessible due to patient confidentiality requirements. Other details obtained from the Crime Scene Department (CSD) included the source of the CO and the type of death (homicidal, suicidal, or non-intentional). According to the General Department of Criminal Evidence, blood specimens of deceased individuals for potential CO poisoning are carried out only when suspecting that the cause of death is related to CO—for instance, if the crime scene contains sources of gas leaks or fires, such as uncontrolled fires in buildings, coal stoves, automobile fires, etc.

Using crime-scene reports, post-mortem assessments of the deceased were performed in the Department of Forensic Pathology. Based on preliminary evaluations by forensic pathologists, blood specimens were collected at autopsy to investigate CO poisoning. Five milliliters of blood were drawn from the radial artery using a 10 mL heparinized syringe and collected in a 10 mL tube. These samples were assigned to the Forensic Toxicology Laboratory for blood gas analysis. Where investigation was postponed, specimens were stored at 4 °C. For blood gas analysis, an average of three measurements was used for analysis.

Automated blood gas analysis was performed in the Forensic Toxicology Laboratory using a compact blood gas CO-oximeter, the GEM^®^ Premier™ 4000 gas analyzer (Instrumentation Laboratory, Bedford, MA, USA). The analysis was conducted according to the manufacturer’s guidelines. The system consists of two components, a fully automated instrument and a disposable cartridge valid for 30 days’ use (GEM^®^ Premier 4000™ PAK). The cartridge contains all of the components necessary for analysis, the electrochemical sensor reagents (lysing and reference solutions for electrochemical measurement), an optical cell for CO-oximetry measurements, a sampling stylus and a waste container. Once a cartridge is installed, an initialization process is required before analysis is performed, namely, a temperature equilibration of the sensor block (40 min), followed by a validation process (20 min). According to the manufacturer’s recommendations, a calibration valuation product test was performed, to ensure the integrity of the newly installed cartridge and the overall system. The operator interacts with the analyzer through a large color touchscreen that displays operational instructions, calibration data and analytical results.

Before analysis, refrigerated specimens were equilibrated at room temperature (23–25 °C) for exactly 15 min. Thereafter, the sample was mixed thoroughly for at least 15 s and analyzed immediately. A single whole-blood specimen of 150 µL was used for the analysis by collecting it in a syringe and introducing it into the analyzer by aspiration. Necessary precautions were taken to expel any air and remove clots or bubbles present in the syringe. The GEM^®^ Premier™ 4000 provides full quantitative analysis within 95 s for up to 21 parameters. It uses different analytical methods depending on the parameters to be analyzed. For the CO-oximetry analysis, the blood sample was chemically hydrolyzed and placed in an optical cell to measure light absorption using a broad-spectrum (475–650 nm) spectrometer. The outputs from this system included measurements of the levels of total Hb, oxygen-carrying Hb, COHb and methemoglobin in a given blood specimen.

Analytical reports were provided to the Forensic Pathology Unit to determine the cause of death. In the current study, we collected and analyzed all laboratory reports that were generated for individuals suspected of having died from CO poisoning from 2014 to 2018 (5 years). Information about the deceased was gathered from the Forensic Pathology Unit, although certain details were inaccessible due to patient confidentiality requirements. Other details obtained from the Crime Scene Department (CSD) included the source of the CO and the type of death, homicidal, suicidal, or non-intentional. In addition, the CSD reports were used to investigate the distribution of fatalities associated with CO poisoning among the six different Kuwaiti governorates, Al Jahra, Hawalli, Al Ahmadi, Al Asimah, Al Farwaniyah and Mubarak Al-Kabeer. GraphPad Prism^®^ version 6 (GraphPad Software, San Diego, CA, USA) and Microsoft^®^ Excel^®^ 2016 (Microsoft Corporation, Redmond, WA, USA) were used for raw-data visualization and graph creation.

## 3. Results

### 3.1. Suspected vs. Confirmed CO Poisoning

**Figure 1:**
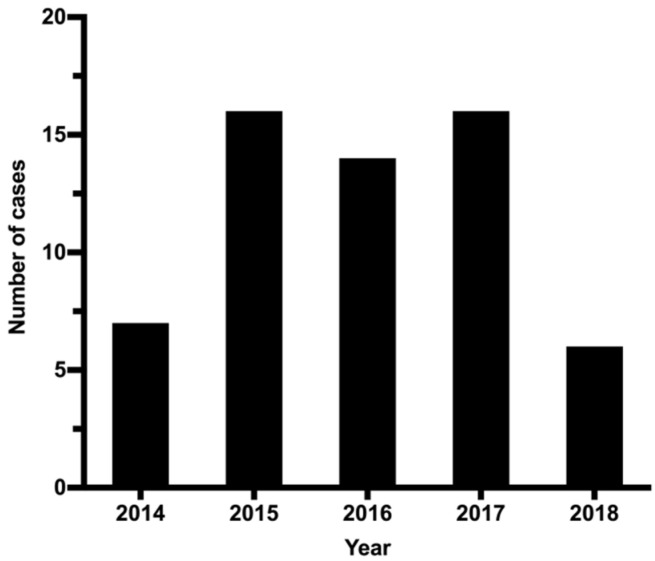
CO poisoning fatal cases (n = 59) in Kuwait (2014–2018).

In total, 203 suspected CO poisoning cases were received and processed from 2014 to 2018 (Table 1), of which 59 cases were confirmed as CO poisoning (Figure 1). In the 5-year period of the study, the COHb% in the blood samples were as follows: <10% in ~44.8% of cases, 10–20% in ~2.4% of cases, 21–30% in ~7.4% of cases, 31–40% in ~6.4% of cases, 41–50% in ~9.9% of cases and >50% in ~29.1% of cases. Generally, COHb% of young casualty is high and low as age progresses. Table 2 shows the average COHb% in the blood samples of the 59 confirmed CO poisoning cases between 2014 and 2018. The 5-year average COHb% was 63.1 ± 8.2%% (range of 50.7–80.1%).

### 3.2. Demographic Features of CO Poisoning

**Figure 2:**
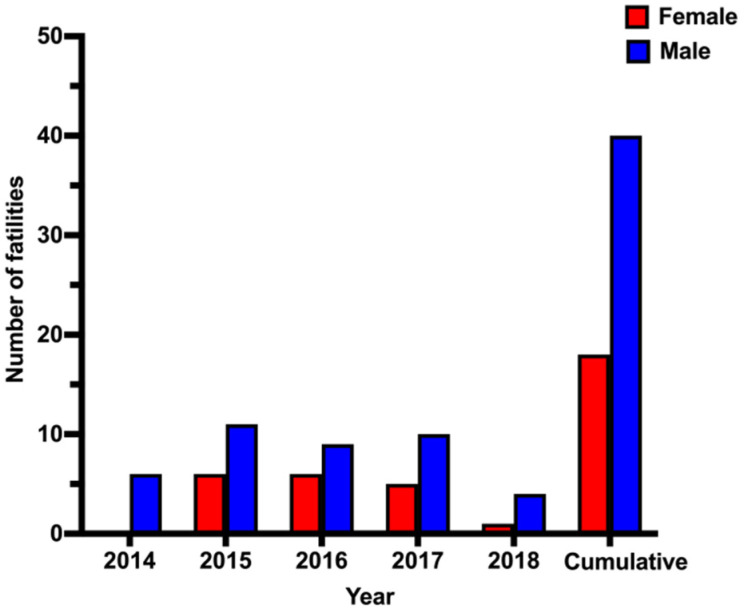
CO poisoning fatal cases (n = 59) by gender (2014–2018).

We analyzed the potential for a link between CO poisoning and the sex of the deceased (Figure 2). Of the 59 confirmed CO-poisoning fatalities, 40 were male and 19 were female. For each year, more males than females died. The highest number of confirmed CO-poisoning fatalities was 16, observed in 2015 and 2017, and the lowest was 6, observed in 2018. We also identified the age groups of those who died due to CO poisoning according to their sex. The 5-year average age for males was slightly higher than females (29.7 vs. 27.4), however, the average age of males and females was somewhat similar (~23–38 years) (Table 3). In 2015 and 2016, the average age of deceased males was higher than that of deceased females; the reverse was true in 2017 and 2018.

### 3.3. Seasonal and Geographical Features of CO Poisoning

**Figure 3:**
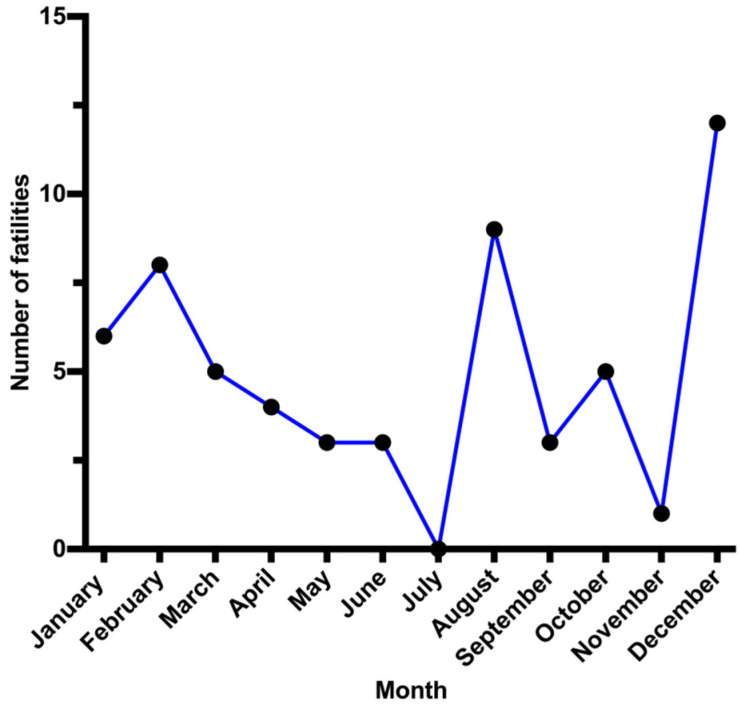
CO poisoning confirmed cases (n = 59) by month (2014–2018).

**Figure 4:**
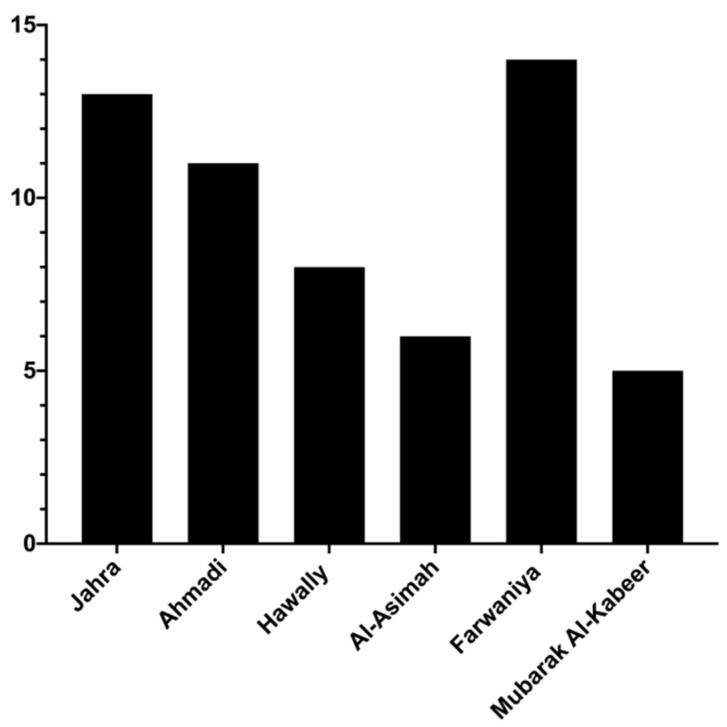
CO poisoning confirmed cases (n = 59) by governorate (2014–2018).

We investigated the potential relationship between CO poisoning and meteorological season. The highest fatality rate associated with CO poisoning occurred during the winter (December–February) (26 cases ~44%). Both spring (March–May) and summer (June–August) had fatality rates of approximately 20% (12 cases) each. During the autumn (September–November), the fatality rate from CO poisoning was the lowest, at ~15% (9 cases) (Figure 3). We also assessed the distribution of CO intoxication-related deaths among the six different governorates of Kuwait. The fatality rate was the highest in Farwaniya (~23%, 14 cases) and the lowest in Mubarak Al-Kabeer (~7%, 4 cases) (Figure 4).

### 3.4. Citizenship and Nationality of CO Poisoning

**Figure 5:**
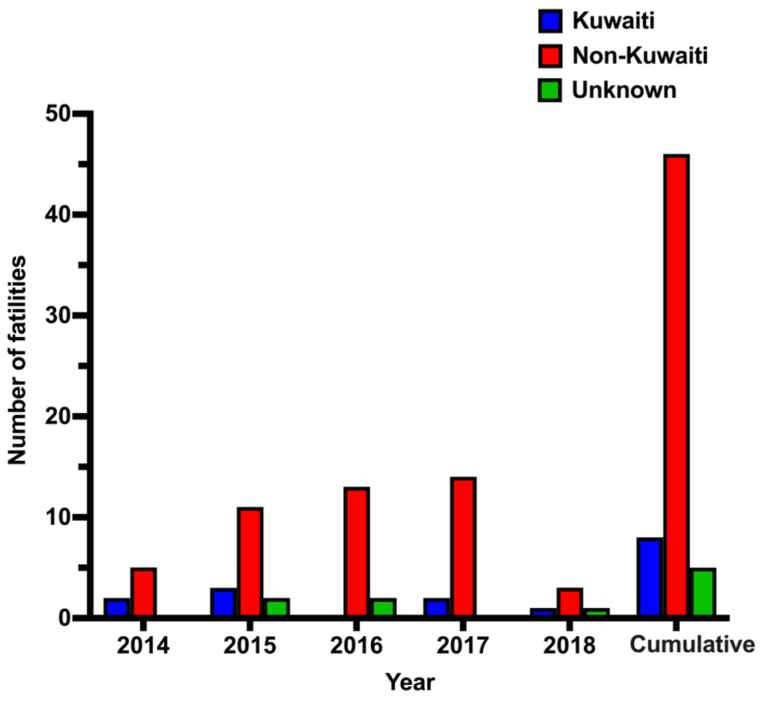
CO poisoning confirmed cases (n = 59) by nationality (2014–2018).

**Figure 6:**
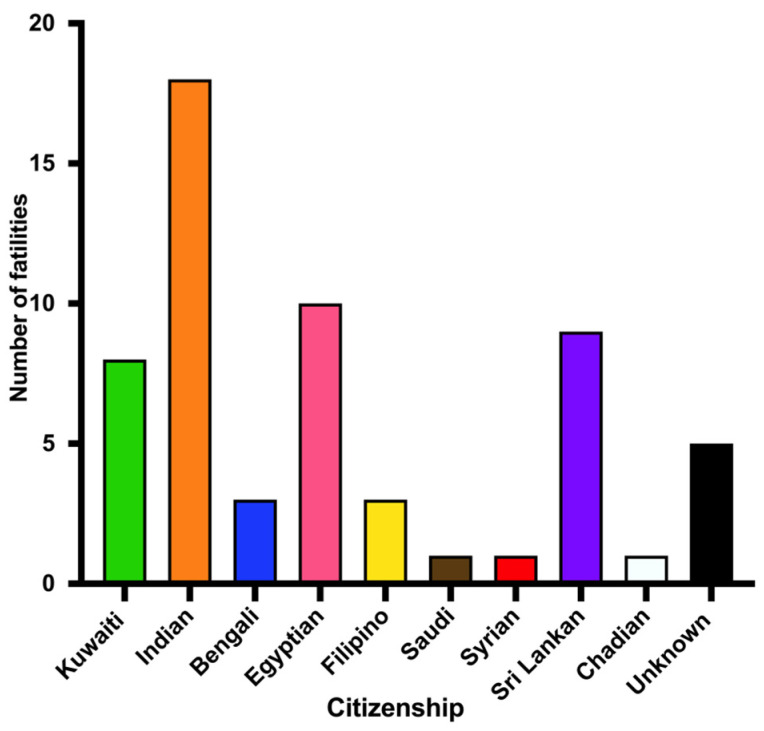
CO poisoning confirmed cases (n = 59) by country of citizenship (2014–2018).

We investigated the CO intoxication mortality rate based on nationality (Kuwaitis and non-Kuwaitis) and country of citizenship (Figure 5). Most fatalities due to CO poisoning occurred among non-Kuwaiti citizens. The number of non-Kuwaiti citizens who died of CO intoxication steadily increased from 2014 to 2017, then declined dramatically in 2018. Furthermore, we examined the country of citizenship of the deceased individuals. The highest rate of mortalities from CO intoxication (~31%, 18 cases) occurred among Indians, followed by Egyptians (~17%, 10 cases), Sri Lankans (~15%, 9 cases) and Kuwaitis (14%, 8 cases) (Figure 6). Many Indians had CO poisoning inside their small, poorly ventilated residential units or tents, while Egyptians constituted the largest percentage of mortality in apartment fires.

### 3.5. Source of CO Poisoning

**Figure 7:**
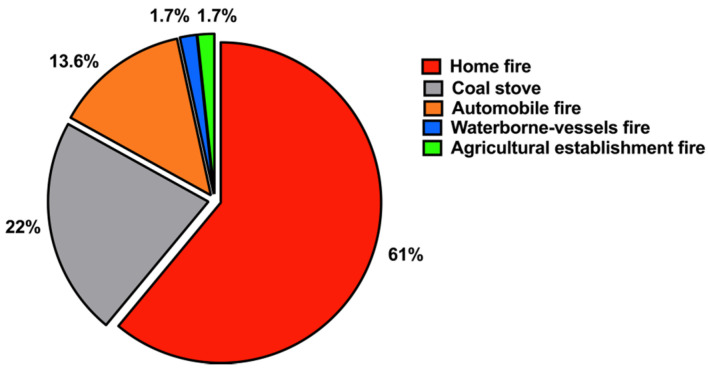
Sources of CO poisoning that led to confirmed cases (n = 59) (2014–2018).

Data analysis showed that 95% of the cases were accidental and 5% were homicidal, with no reported suicides by CO poisoning. The sources of CO in cases of fatal intoxication were as follows: uncontrolled home fires caused by a heat source that is used to heat rooms (e.g., electric furnaces, portable heaters) (61%), coal stoves/charcoal (22%), automobile fires (14%), waterborne-vessel fires (2%) and fires at agricultural establishments (2%) (Figure 7).

## 4. Discussion

The aim of this study was to investigate the prevalence of CO poisoning-associated mortalities occurring in Kuwait over five years (2014–2018) using official police data. To our knowledge, this is the first study that explored CO poisoning exposure and mortality in Kuwait.

CO poisoning is a dose-dependent process by which symptoms of intoxication worsen as the concentration of COHb increases in the blood [14,15]. CO has a high affinity for Hb (~250-fold greater than O_2_) [3]. Upon binding, CO displaces O_2_ from Hb, thereby reducing the oxygen-carrying capacity of the erythrocytes, resulting in hypoxia. Thus, tissues and organs with a high demand for oxygen, including the brain and heart, are affected negatively due to an inadequate oxygen supply [16]; this is the primary mechanism of CO toxicity [2]. Similarly, CO decreases O_2_ storage in muscle cells by binding to and displacing O_2_ from the heme groups of myoglobin [5]. Additionally, CO causes mitochondrial dysfunction by binding to the heme groups of COX [17]. The formation of CO–COX complexes slows down oxidative phosphorylation reactions in the mitochondria and, consequently, reduces energy production [3,16]. Moreover, CO intoxication activates inflammatory signaling pathways, which induce neurological and cardiac injuries. CO facilitates the formation of platelets by displacing nitric oxide from their heme groups, which ultimately leads to the generation of reactive oxygen species and activates different inflammatory pathways [3].

The data used in this study were obtained from the General Department of Criminal Evidence, Ministry of Interior of Kuwait, using samples collected and analyzed during the period 2014–2018 by the Departments of Forensic Pathology and Forensic Toxicology, following the laws on disclosing crime scene autopsy report. Unfortunately, the final cause of death was not among the information collected. The information released regarding the cause of death was the type of death (homicidal, suicidal, or non-intentional). Other information recorded in the reports included the name of the deceased, nationality, age, gender, governorate location, COHb% and the source of CO (e.g., uncontrolled open fire in homes, automobile fire (many of which occur from severe car crashes), coal stoves/charcoal used for cooking or heating, etc.). Access to death certificates on which COHb levels were measured was not obtained. All suspected autopsies of CO poisoning were sampled, that is, a total of 203 cases. According to the General Department of Criminal Evidence, COHb levels of blood specimens were tested for CO poisoning only when suspecting that the cause of death was associated with potential CO poisoning—for instance, if the crime scene contains suspected heat sources, such as coal stoves, candles, gas leaks, etc. In some cases, incomplete combustion from coal stoves that are used during desert camps resulted in the death of children and whole families while asleep.

COHb has been reported to range from 2–98% in deaths from CO, averaging 66% [7]. The highest reported survival level was 73%, with COHb at 70% as a barrier for survivability; COHb 98% was the highest level reported in the literature [7]. Out of 100 patients with acute myocardial infarction, the mean COHb level was 55 ± 6%, of which 51 patients had COHb levels over 55%; three patients with COHb levels of 71%, 71% and 73% survived 56, 56 and 62 months, respectively [18,19]. Nationwide, in patients with CO poisoning referred for hyperbaric oxygen treatment, the average initial COHb was 23.4 ± 10.4% (range of 0.1–77%). COHb is a marker of recent exposure to CO and not an indicator gauge of CO poisoning severity. In the current study, we related the fatal concentration for CO poisoning to be COHb at 50% or more, based on previous studies [20,21]. The median level of COHb in people dying of uncomplicated CO poisoning was reported to be 53–55% [20] and 50% or more of COHb in the blood is an indicator of fatality [21]. Only rarely do people live with COHb levels greater than 50% [18]. Although some people may die with lower levels of COHb, we only selected the confirmed cases as suggested by the pathologists. Out of 203 suspected cases, 22 cases had missing COHb values, 67 cases had COHB levels ≤ 10%, 55 cases had COHb levels in the range from >10% to <50% and the remaining (59 cases) had COHb ≥ 50%. We only analyzed the cases with COHb ≥ 50% which the pathologists confirmed as CO poisoning deaths.

In the present study, 203 samples tested positive for COHb. The level of COHb ranged between 0.3% and 80.1%. We deemed a COHb% of ≥50% as confirmation of CO-poisoning with a five-year average COHb% of 63.1 ± 8.2% (range of 50.7–80.1%). Using this criterion, we confirmed that 29% of the investigated cases were due to CO poisoning. The five-year cumulative mean of COHb% in the blood samples of cases was ~63%. The level of COHb in the blood is influenced by several factors including, age, sex, Hb level and the degree and duration of CO exposure [22].

Overall, there was a clear sex difference in CO-related mortality. The mortality rate among males (68%) was more than double that among females (32%). A similar male predominance has been reported in other published studies in the Middle East [11,13,23]. Such sex-based differences cannot be explained with available data. Further investigations of factors, such as economic status, lifestyle, behavior and adherence to safety protocols, will be necessary.

Our analysis showed that male and female CO-poisoning victims had similar mean ages (males, 24–38 years; females, 23–38 years). This age range is consistent with that of Rahimi et al. (2019) (18–37 years), who reported fatal cases of CO poisoning in Iran [23]. Previous studies have revealed that the COHb concentration at the time of death is usually higher in younger victims than the elderly [24,25,26]. It should be pointed out that young adults (20–39 years old) represent 32% of the general population in Kuwait, according to the latest data published by the national Central Statistical Bureau [27]. Therefore, by chance, CO-poisoning mortality is expected to be common among young adults. The largest number of deaths (~23%) occurred in Farwaniya Governorate, which comprises many expatriates living in small residential units.

Deaths from CO intoxication in Kuwait occurred most often (26 cases ~44%) during the winter (December–February). This result correlates with other reports, with deaths related to CO poisoning being more common in cold weather [11,13,23,28]. This phenomenon may be explained using various methods used to maintain a warm indoor environment, including gas heating appliances, coal heaters and stoves and gasoline-powered electric generators, all of which produce CO due to incomplete combustion [29]. Although a peak of CO poisoning was also reported in August (9 cases), five cases were from s single family.

The mortality rate among expatriates outnumbered that among Kuwaiti citizens. This can be explained by the fact that foreigners represent approximately 70% of Kuwait’s total population [27]. The leading nationality among CO-related fatalities was Indian, which corresponds to Kuwaiti’s largest group of expatriates. Many expatriates live in small residential units with improper safety measures. Open flame and gas cookers are very common and large amounts of combustibles (curtains, furniture, etc.) constitute fire hazards. Expatriate family members, including many children, were victims of fires in small apartments. Burning charcoal in coal stoves for cooking or warming indoor environments in poorly ventilated housemaid rooms or camping tents was associated with CO poisoning.

Intentional suicidal CO poising was not reported in this study. In a recent study of patterns of suicide in Kuwait, there were 297 cases of suicides, from 2014 to 2018, of which 90% were related to hanging, while no case was associated with CO poisoning [30]. In another study, there were 347 suicidal cases, of which 60% were related to hanging, 17% to sharp objects and 14% to drinking poison [31].

CO poisoning may result from negligence in observing safety regulations, lack of personal awareness of the potential risk of CO poisoning and low socioeconomic status [32]. Interestingly, our results demonstrated that smoke inhalation from uncontrolled home fires caused by a heat source was responsible for most CO poisoning fatalities (~61%), followed by coal stoves/charcoal (22%). This result is supported by those of several other studies [13,14,33]. Such a source of CO intoxication is preventable and depends on avoidance and early detection [29]. The large number of fire-related CO poisoning in Kuwait reported in this study is most likely due to the lack of adequate fire-safety regulations in residential settings, such as those regarding the installation of smoke alarms and CO detectors.

## 5. Conclusions

The prevention of CO poisoning depends on the following factors: (1) source reduction, (2) early detection and (3) increased public awareness of the dangers of CO poisoning. In many cases, CO poisoning can be attributed to faulty equipment or a lack of awareness of the associated risk. Practical measures can be applied to reduce the risk of CO poisoning, including proper installation and regular maintenance of home-heating appliances and appropriate ventilation during the use of butane and kerosene heaters or coal stoves/charcoal. Based on our findings, we propose that the local government should endorse related health education, informing the local population about the potential risks of CO poisoning, emphasizing prevention. Public-health interventions to improve health and safety behaviors may prevent a large number of CO poisoning cases. This includes (1) increasing public awareness regarding the importance of regular assessment and maintenance of heating and cooking appliances, (2) mandating the installation of smoke alarms and CO detectors in residential settings and (3) education regarding the proper use of portable equipment that generates CO (gasoline-powered generators and coal stoves). Additionally, identifying and targeting population-specific subgroups at higher risk of CO-related mortality is essential to guide educational efforts.

A vital strength of the present study is the validity of the data, as data were obtained from an authorized database of the Government of Kuwait. Nonetheless, this study was limited by the absence of information about the dose of CO exposure, in terms of amount, duration and severity. Exposure dose is a critical factor affecting the outcome of CO poisoning incidents. Although the parameters measured in this study are objective biomarkers of CO exposure, the exposure dose cannot be estimated accurately based on COHb concentration without information on the duration of exposure. Thus, the dose-response correlation could not be evaluated in this study and such investigation should be performed in the future.

## Figures and Tables

**Table 1:** Average COHb% in blood specimens of all CO poisoning cases (n = 203) by age groups in Kuwait.

Year		Age							
	n	0–9	10–19	20–29	30–39	40–49	50–59	60–69	70~
2014	36		20.95	11.2	35.1	32.1	0.75		
2015	43	54.8	25.9	31.2	38.2	39.1	3.0	2.1	
2016	45	32.3	35.5	37.8	26.8	47.3	25.6	2.0	1.7
2017	46	28.4	42.8	37.6	42.6	43.8	8.1	0.4	1.3
2018	33	20.0	2.3	39.0	30.2	20.9	5.4		

**Table 2:** COHb% in blood specimens of CO poisoning fatal cases (n = 59) in Kuwait.

Year	n	Avg. COHb%	SD	Range (min–max)
2014	7	66.6	8.8	56.2–76.3
2015	16	63.9	8.6	50.9–75.8
2016	14	59.9	7.6	50.7–79.7
2017	16	64.6	8.5	51.6–80.1
2018	6	61.2	6.2	51.5–66.1
	59	63.1	8.2	50.7–80.1

**Table 3:** CO poisoning confirmed cases (n = 59) by age (2014–2018).

Year	Male			Female		
	Avg	SD	Range (min–max)	Avg	SD	Range (min–max)
2014	38	6.5	30–46	-		
2015	24	13.5	3–41	23	18.8	2–45
2016	33	14.1	2–50	28	20.5	6–54
2017	28	11.1	4–42	31	7.2	21–40
2018	30	5.5	25–36	38	-	-
Avg	29.7	12.0	2–50	27.4	15.7	2–54

## Data Availability

All data is available upon request (original data is in Arabic language).
